# Large Language Model–Generated Patient Instructions for Prescriptions in Primary Health Care: Preclinical Algorithm Validation

**DOI:** 10.2196/84444

**Published:** 2026-05-26

**Authors:** Zilma Silveira Nogueira Reis, Elisa Tuler Albergaria, Adriana Silvina Pagano, Eura Martins Lage, Flávia Ribeiro de Oliveira, Cristiane dos Santos Dias, Juliana Almeida Oliveira, Gláucia Miranda Varella Pereira, Isaias Jose Ramos de Oliveira, Érico Franco Mineiro, Igor Carvalho Lima Oliveira, Davi dos Reis de Jesus, Antônio Pereira de Souza Júnior, Igor de Carvalho Gomes, Rodrigo André Cuevas Gaete, Ricardo Cruz-Correia, Leonardo Rocha

**Affiliations:** 1 Health Informatics Center Universidade Federal de Minas Gerais Belo Horizonte Brazil; 2 Computer Science Department Universidade Federal de São João Del Rei São João Del Rei Brazil; 3 Arts Faculty Universidade Federal de Minas Gerais Belo Horizonte Brazil; 4 Department of Physiotherapy, School of Physical Education, Physiotherapy and Occupational Therapy Universidade Federal de Minas Gerais Belo Horizonte Brazil; 5 School of Architecture Universidade Federal de Minas Gerais Belo Horizonte Brazil; 6 National Secretary of Primary Care of the Brazilian Ministry of Health Brasilia Brazil; 7 MEDCIDS Porto University Porto Portugal

**Keywords:** primary health care, drug prescriptions, large language models, generative artificial intelligence, digital health

## Abstract

**Background:**

The application of generative artificial intelligence to simplify medication use instructions has the potential to enhance people’s health by improving treatment adherence.

**Objective:**

We evaluated the performance of large language models (LLMs) in generating medication usage instructions to complement prescriptions in primary health care.

**Methods:**

This randomized, blinded experimental preclinical study used prescription-inducing scenarios, assigned to 62 health care professionals, to validate instructions generated by LLMs during electronic prescriptions. The instructions were generated by ChatGPT-4.0 (OpenAI), Llama3.1-8B (Meta), and Llama3.1-8B-RAG (Meta) using retrieval-augmented generation based on patient information leaflets. Performance metrics assessed adequacy, completeness, clarity, language simplification, usefulness, and errors in the generated instructions, with scores to analyze overall and individual metrics.

**Results:**

The 3 models yielded high overall scores for producing qualified instructions (ChatGPT-4.0: median 88.4, IQR 22.8; Llama3.1-8B: median 66.5, IQR 50.9; Llama3.1-8B-RAG: median 79.9, IQR 34.4; Kruskal-Wallis test *P*=.003). Llama3.1-8B-RAG received evaluations with similar overall scores to ChatGPT-4.0 (post hoc test, *P*=.05) and similar to Llama3.1-8B (post hoc test, *P*=.44). ChatGPT-4.0 outperformed Llama3.1-8B (Bonferroni test, *P*<.001). Regarding specific domains, Llama3.1-8B-RAG received scores equivalent to those of ChatGPT-4.0 for adequacy (mean 6.24, SD 2.3 vs mean 6.82, SD 2.1; post hoc test, *P*=.54); completeness (mean 5.94, SD 2.2 vs 6.55, SD 1.9; post hoc test *P*=.38), clarity (mean 5.77, SD 2.4 vs mean 6.68, SD 1.9; post hoc test *P*=.09), and usefulness (mean 5.42, SD 2.4 vs mean 5.96, SD 2.2; post hoc test *P*=.63). ChatGPT-4.0 received higher scores in the language simplification criterion than Llama3.1-8B-RAG (mean 7.05, SD 1.5 vs mean 5.44, SD 2.6; post hoc test *P*<.001). Interrater variability in assigning scores ranged from 4.2% (n=3) to 85.8% (n=6) among primary health care professionals. Instructions leading to incorrect use of the medication had similar frequency among the models(ChatGPT-4.0: n=15, 22.7%; Llama3.1-8B: n=19, 22.8%; Llama3.1-8B-RAG: n=19, 22.8%; chi-square test *P*=.71). The frequencies of hallucination were similar (ChatGPT-4.0: n=7, 10.6%; Llama3.1-8B: n=9, 13.6%; Llama3.1-8B-RAG: n=6, 9.1%; chi-square test *P*=.67).

**Conclusions:**

The open-source LLM enhanced with external information presented similar performance to the closed-source model, except for ChatGPT4.0, which was superior in language simplification of messages. LLM generation demonstrated potential for instructing patients on medication use. Nonetheless, the introduction of this innovation into the electronic prescribing workflow demands prescriber validation for human oversight of the technology and requires a strategy for LLM performance governance.

**International Registered Report Identifier (IRRID):**

RR2-10.12688/verixiv.1359.1

## Introduction

The association between medication nonadherence and a higher prevalence of diseases demonstrates that not using, or incorrectly using, prescribed medications represents a burden for both patients and health care systems [1]. In contrast, investing in processes that can promote treatment adherence is a strategy capable of mitigating excessive and avoidable costs incurred by misuse, thereby promoting the health of individuals, especially those with chronic conditions. The underuse of prescribed medications, whether intentional or unintentional, is considered a major global public health problem. One study examined 195,930 electronic prescriptions in Massachusetts, United States, to analyze the drug classes most associated with primary patient nonadherence immediately after the first prescription, with findings for medications for diabetes (31.4%), arterial hypertension (28.4%), and hyperlipidemia (28.2%) [2]. In low- and middle-income countries, the estimated occurrence of nonadherence is even higher, potentially reaching 50% of prescriptions [3]. Health care scenarios with limited resources also face significant challenges in controlling noncommunicable chronic diseases, mental disorders, tuberculosis, and HIV infection, further emphasizing the importance of providing measures that favor treatment adherence [3].

Technological interventions facilitated by digital technology have the potential to expand the coverage and quality of health services [4]. In primary health care (PHC), which serves as the entry point for individuals into the health systems, digital transformation involves introducing useful, widely applicable, and cost-effective technological resources. It is also essential that these systems are developed to meet the needs of individuals and communities [5]. One successful example of digital systems in health care is electronic prescribing (e-prescribing). The incorporation of a standardized medication menu with informed dosages, maximum and minimum limits, administration routes, and alerts for drug interactions, in addition to mitigating readability issues in prescriptions, proved useful by reducing preventable medication errors by 55% [6]. In PHC settings, the possibility of direct electronic communication between the prescribing professional and the dispensing professional is also a point in favor of benefits such as increased safety and quality of care provided [7].

Despite advances in e-prescribing, medication adherence remains a barrier to effective treatment. The lack of personalized treatment, customizing medication use instructions to patients’ educational level, culture, and health literacy, has been identified as a barrier to adherence [7]. Addressing the diverse living conditions and health needs of individuals seeking primary care goes far beyond simply replacing handwritten prescriptions with e-prescribing systems. Scientific advancements demonstrate the potential of generative artificial intelligence (AI) to support health care professionals and improve people’s health. Among these is the promise of using intelligent systems to help patients correctly use prescribed medications [8]. However, the adoption of AI solutions remains challenging, encompassing regulatory aspects, infrastructure investments, the need for transparency and governance, as well as resistance to change among health care professionals [9].

In Brazil, the Unified Health System (SUS) features a standardized and interoperable electronic health record system for PHC, named e-SUS PHC. Implemented in 2011 and developed collaboratively ever since by the Secretariat of Primary Health Care of the Ministry of Health, with participation from health care professionals, health managers, and universities [10], the system includes a specific functionality for e-prescribing [11]. The national reach of e-SUS Primary Health Care (e-SUS APS) and its widespread use by Brazilian basic health units generate a vast quantity of daily electronic health records, related to approximately 1.48 million consultations per day, according to January 2025 data from the system’s coordination, authors RACG and ICG [11].

The personalization of patients’ instructions on medication use, drawing on large language models (LLMs) and based on prescription data, has been studied using the computational structure of e-SUS APS [12]. Preliminary results demonstrated the potential of LLMs in generating instructions to facilitate communication between health care professionals and patients, which could promote greater adherence to treatments [12]. The development process has been conducted collaboratively between academia and the Brazilian Ministry of Health—General Coordination of Innovation and Digital Acceleration of the Secretariat of Primary Health Care—through an interdisciplinary team of researchers in a simulated environment that represents the most frequent real-world prescription scenarios in PHC. Regulatory requirements and principles for the ethical and responsible use of AI have been considered since the planning stage. An initial version of the product was made available for testing, capable of incorporating additional instructions into prescriptions generated within the electronic system, on demand and at the free choice of health care professionals [13].

Building on the progress achieved in developing this innovative product, this study aims to evaluate the performance of LLMs, adjusted to citizens’ needs, as perceived by users of the e-SUS PHC. This represents a relevant step for planning the nationwide implementation of a new functionality within the e-SUS APS electronic health record.

## Methods

### Overview

The methodology followed international guidelines for publishing studies evaluating Health Informatics apps, STARE-HI (Statement on Reporting of Evaluation Studies in Health Informatics) [14]. The technical artifact under evaluation is the use of LLMs as a functionality within an e-prescribing system, the e-SUS PHC, and its usefulness for patients, as perceived by the prescribing health care professional.

### Study Design

This is a randomized, blinded experimental study that evaluated the quality of instructions generated by 3 language models designed to complement medication prescriptions during the preparation of e-prescriptions.

### Development of the Prescriber Support Solution

Using the collaborative methodology of the Scrum framework within Agile methodology, regular meetings facilitated the progressive development of automated intelligent solutions, adjusted to the e-SUS PHC system, according to the national electronic health record environment. Based on a review of the state of the art and partial validation processes, the software has received continuous adjustments, as presented in Table 1, which describes the development stages.

**Table 1:** Stages of innovation development for the generation of medication use instructions with the support of generative artificial intelligence.

Milestones	Achieved or planned results
Workshop for interdisciplinary conceptual alignment	Multidisciplinary research team combining expertise in health sciences, data science, design, and linguistics, with proven capacity for development, evaluation, and refinement of the proposed innovation.
Selection of LLMs^a^	Completion of feasibility, safety, and explainability analysis of available LLMs, and selection of models for testing [15].
Development of a simulated database comprising PHC^b^ clinical histories	A set of 104 clinical scenarios involving citizens with different characteristics, with expected medication use instructions prepared by specialists, already reported [12].
Development of a virtual testing environment	An interface similar to the e-SUS PHC^c^ prescribing environment, which presents citizen data, one medication prescription at a time, reference instructions, and usage instructions generated by models with a form containing evaluation metrics.
Proof of concept with internal evaluation conducted by the research team	Comparative evaluation of the performance of ChatGPT-4.0 and Llama3.1-8B models fine-tuned through prompt engineering. The study demonstrated LLM potential to generate supplementary usage instructions to accompany medication prescriptions, already reported [12].
Model enhancements	Introduction of RAG^d^ into models to mitigate inadequacies in medication use instructions generated by LLMs, already reported [13].
Refinement of the simulation environment for real-time testing (this study)	Interface adjusted for health care professionals to elaborate electronic prescriptions using LLMs, generating instructions on how to use the medication.
External validation of minimum product version (this study)	Use of virtual simulation environment by prescribers within the e-SUS APS^e^ system. Comparative evaluation of the performance of models based on ChatGPT-4.0 and Llama3.1-8B, fine-tuned through prompt engineering, tested with and without RAG.
Responsible AI^f^	Analysis of requirements for transparency, robustness, usability, traceability, universality, and fairness necessary for implementation of the innovation in e-SUS APS, following international consensus criteria of Lekadir et al [9].
Planning deployment and implementation	Planning national implementation of a new functionality in the Brazilian e-SUS PHC electronic health record. Next steps.

^a^LLM: large language model.

^b^PHC: primary health care.

^c^e-SUS PHC: an electronic health record system for PHC of the Unified Health System in Brazil.

^d^RAG: retrieval-augmented generation.

^e^e-SUS APS: e-SUS Primary Health Care.

^f^AI: artificial intelligence.

### Panel of Expert Evaluators

A panel of health care professionals was invited to voluntarily participate as human evaluators of instructions generated by the language models. Eligibility criteria included being a professional working in the national health care system—the SUS—preferably using the e-SUS PHC electronic health record, and holding legal authority to prescribe medications conferred by a professional council, which in PHC settings includes physicians, dentists, and nurses.

Email invitations were sent with the support of municipal PHC managers through the PHC Secretariat, totaling 1111 correspondences across different Brazilian municipalities, with a minimum of 60 volunteers as the target, since there is no established consensus on sample size for generative AI model validation with end users. Given the exploratory and semantic nature of LLM output, the theoretical saturation criterion was more appropriate than a priori sample probabilistic calculation [16]. The first step was giving informed consent and assent through the online questionnaire. A total of 134 health care professionals formally consented and received basic training on using the simulation environment for LLM testing, which included textual and audiovisual explanations, virtual meetings, and a permanent communication channel for inquiries. Of the 134 health professionals who consented, 72 (53.7%) did not complete any evaluations. The final panel of evaluators consisted of 62 specialists, of whom 61 completed the full evaluation process as proposed, while 1 completed most of it, meeting the saturation criterion, achieving all the main categories of flaws that the models present. The form used to characterize the evaluator is found in Table S1 in Multimedia Appendix 1.

**Figure 1:**
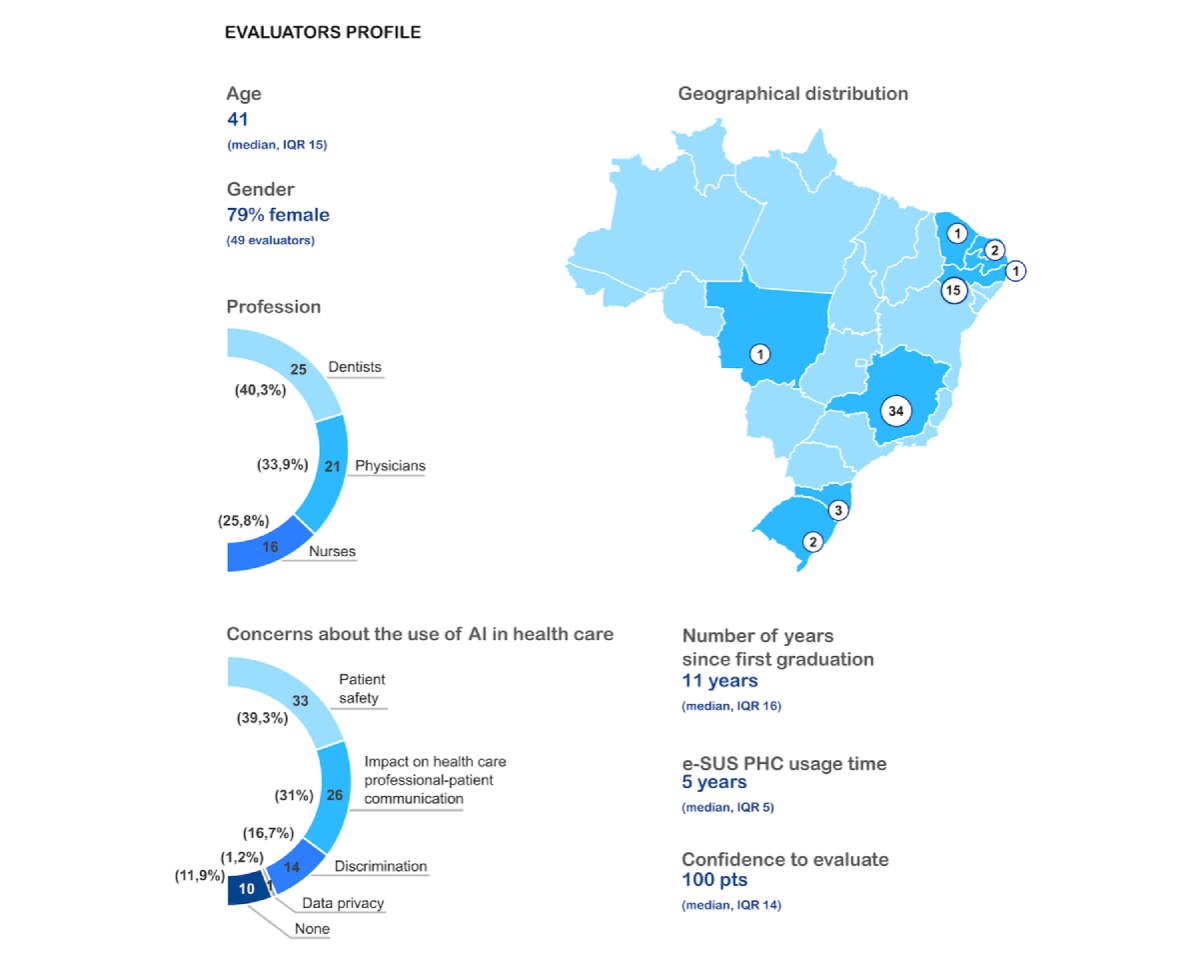
Profile of health care professionals panel and their evaluation of texts generated by large language models. AI: artificial intelligence; e-SUS PHC: an electronic health record system for PHC of the Unified Health System in Brazil.

**Figure 2:**
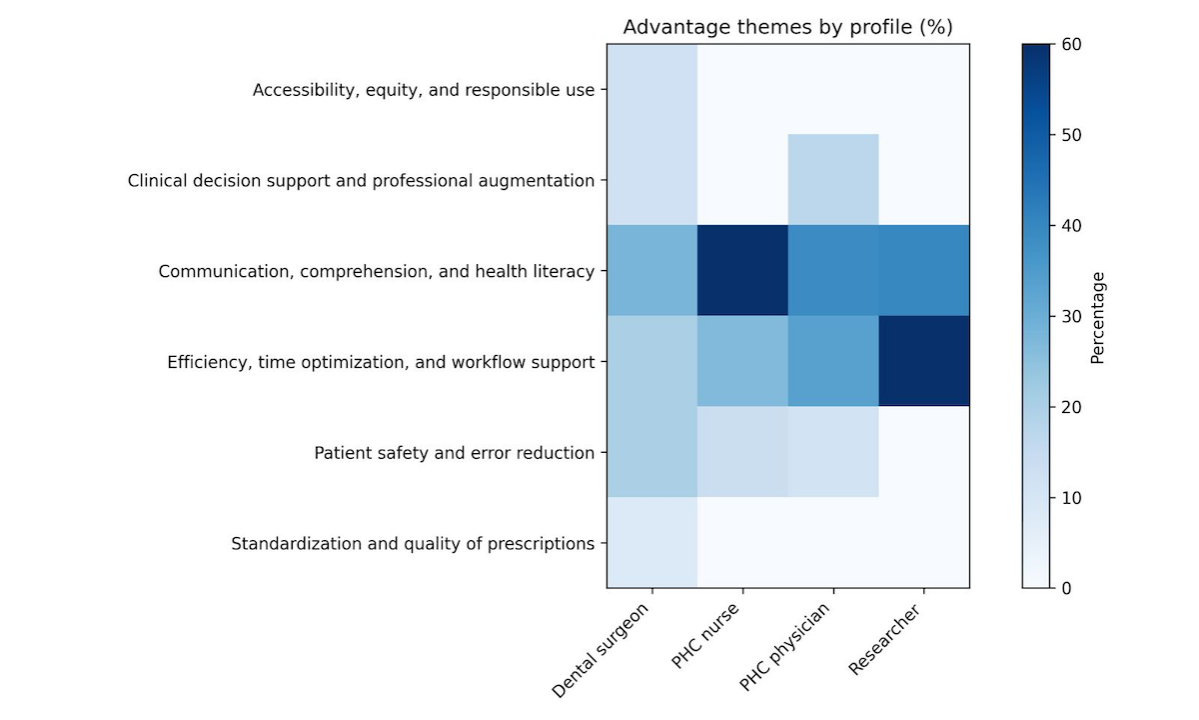
Advantages of using artificial intelligence to support communication between health professionals and patients. PHC: primary health care.

**Figure 3:**
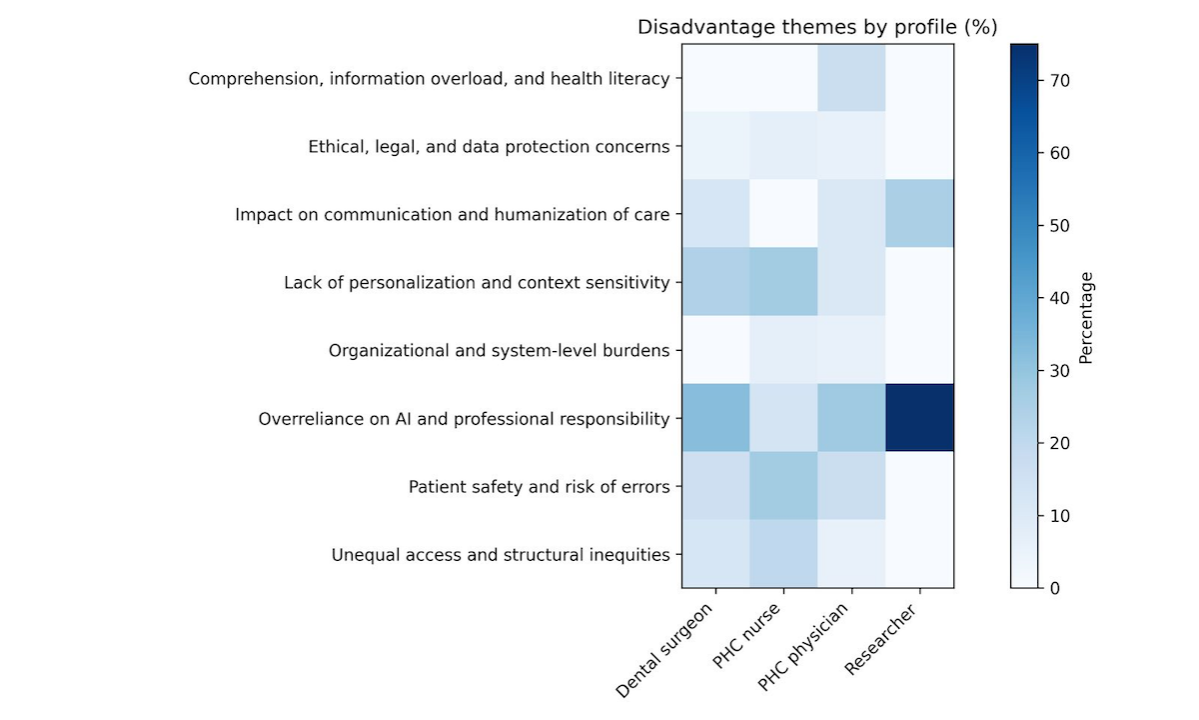
Disadvantages of using artificial intelligence to support communication between health professionals and patients. AI: artificial intelligence; PHC: primary health care.

For each prescription, health care professionals self-reported their confidence in evaluating the scenario, ranging from 0 to 100, stating their perceived knowledge necessary to assess that specific prescription case. The evaluators’ confidence had a median of 100 (IQR 14), with 79.3% (157/198) of evaluators having confidence of >80% and 68.2% (135/198) having confidence of 90%. Figure 1 presents sociodemographic data of evaluators, with a median of 5 (IQR 5) years of experience in the SUS PHC system, and mostly female health professionals. After the evaluation phase, professionals were invited to share their opinions and concerns regarding the use of AI in health care. Two open-ended questions were used to assess evaluators’ opinions on the advantages and disadvantages of using AI to support communication between health professionals and patients. Participant statements were first thematically grouped into a set of overarching advantage and disadvantage categories reflecting major concerns about AI-assisted generation of medication use instructions. Advantage themes included patient safety and error reduction; communication, comprehension, and health literacy; efficiency, time optimization, and workflow support; clinical decision support and professional augmentation; standardization and quality of prescriptions; accessibility, equity, and responsible use. Disadvantage themes included patient safety and risk of errors; overreliance on AI and professional responsibility; lack of personalization and context sensitivity; impact on communication and humanization of care; comprehension, information overload, and health literacy; ethical, legal, and data protection concerns; organizational and system-level burdens; unequal access and structural inequities. Each statement was then manually coded using this category framework, allowing multiple categories to be assigned when relevant. For visualization, thematic codes were aggregated by professional profile and polarity (advantages vs disadvantages), and frequencies were normalized as within-profile percentages of coded themes. Because individual statements could be associated with more than one theme, percentages do not sum to 100% within profiles. The resulting distributions are shown in the heatmaps in Figures 2 and 3, where color intensity represents the relative prominence of each theme within dental surgeons, physicians, nurses, and researchers, with darker shades indicating higher thematic salience. In Figure 1, we present the evaluators’ profile and geographic distribution. Figures 2 and 3 present heatmaps of advantage and disadvantage themes, respectively, concerning the use of AI-generated medication instructions to support communication between health professionals and patients across the 4 professional profiles.

### Evaluation Workflow of LLMs

The evaluation workflow for instructions generated by the models encompassed the following sequential steps: (1) development of the simulation environment containing simulated clinical scenarios, (2) definition of model validation metrics, and (3) data collection from health care professionals’ evaluations.

#### Minimum Product Version

To provide a realistic e-prescribing experience supported by LLMs, a simulation environment was developed with a layout similar to that of the e-SUS PHC electronic health record. A set of 16 clinical scenarios, contextualized within primary care practices, was provided by the researchers and internally validated for clarity and relevance to the intended context, with each scenario requiring only one medication. Ten scenarios were designed to induce medical prescriptions, 3 for nursing prescriptions, and 3 for dental prescriptions. To enable health care professionals to make choices and decisions based on their competence and prior experience, the environment provided a list of medications containing the active ingredient name, presentation form, concentration, and administration routes, compiled from the e-SUS PHC electronic health record. The detailed information prepared to generate prescriptions in the virtual validation environment is available in Tables S2-S4 in Multimedia Appendix 1.

Three models were compared: ChatGPT-4.0 (OpenAI), Llama3.1-8B (Meta), and Llama3.1-8B with on-demand RAG (retrieval-augmented generation; Meta) using patient information leaflets (PILs) from the National Health Surveillance Agency (ANVISA). Using 16 scenarios, LLMs generated instructions that gathered 48 test options. We used the same prompt engineering, designed and previously tested on another experimental database from our product’s earlier development stage, available in our previous publication [13]. The models’ temperature parameter was set to 0 for ChatGPT-4 and 0.00001 for Llama 3 to increase the predictability of the generated texts. Input variables, prompts used, and the RAG resource are described in Table S5 in Multimedia Appendix 1.

#### Evaluation Criteria

The performance of the 3 models in generating text-format outputs to facilitate medication use was analyzed according to criteria for validating generative AI models recommended by Tam et al [17]. The criteria considered were as follows:

1. Adequacy and correctness: model’s capacity to generate responses that are accurate and appropriate to the user’s purpose, context, and needs.
2. Completeness: model’s capacity to generate responses or content that adequately covers all relevant aspects of a query or task.
3. Clarity: model’s capacity to present information in an understandable, organized, and unambiguous manner.
4. Language simplification: model’s capacity to generate tailored instructions that can engage and be understood by users, such as addressing them by their first name, using commands (imperative verb forms), and simplifying technical content introduced by RAG in PILs.
5. Usefulness: model’s capacity to generate medication use instructions that are useful for complementing prescriptions.

A free-text field was provided for the evaluator to complete their assessment. The evaluation form is available in Table S6 in Multimedia Appendix 1.

Each criterion was evaluated by users on a 5-point scale (1: strongly disagree to 5: strongly agree), which was adjusted to a range of 1 to 5. The normalization of the scale for a comparable sum from the 1-5 Likert scale is adjusted as follows: for odd items (positive), the adjustment is “response - 1” (ie, 1 becomes 0 and 5 becomes 4); for even items (negative), the adjustment is “5 - response” (ie, 1 becomes 4 and 5 becomes 0). For instance, when an evaluator assigned 5 for positive and 1 for negative in adequacy, the raw score was calculated as follows: [(*5*–1) + (5–*1*)] = 8. Responses in italic.

The performance criteria for the models were weighted according to their clinical relevance, as determined by the researchers: adequacy received a weight of 2, clarity and usefulness received a weight of 1.5, and completeness and language simplification each received a weight of 1. Table 2 presents details regarding the composition of the overall score assigned to each question of the evaluation instrument and its weighting.

The weighted overall score generated an increasing numerical indicator, ranging from 0 to 56 (sum of total achievement), while the score per criterion received a value according to its weighting. Normalized to 100, the value is calculated by the formula: performance = 100 × weighted score/56. For instance, when an evaluator assigned 5 for positive and 1 for negative in adequacy, clarity, usefulness, completeness, and for language simplification, the weighted overall score was calculated as follows: [(5–1)+(5–1)]+[(5–1)+(5–1)]+[(5–1)+(5–1)]+[(5–1)+(5–1)]+[(5–1)+(5–1)]=40. In this example, applying the domain weights, the weighted score is 2×8+1.5×8+1.5×8+1×8+1×8=56, and the performance was 100×56/56=100.

There are fundamental advantages in the proposed methodology derived from the System Usability Scale [18]: (1) the balance between alternating positive and negative statements, instead of strictly positive propositions, aims to avoid the respondent’s tendency to agree and allows for capturing more precise nuances of user perception. (2) The adaptation of the sum allows for generating a total normalized score from 0 to 100—more intuitive and easier to interpret (100=excellent quality, 0=extremely low quality). We calculated the internal consistency of the scale and interrater variability.

Additionally, errors in the texts generated by the models were verified by health care professional evaluators. If present, they were classified according to a typology proposed by Roy et al [19] for ChatGPT, simplified into 4 types: error type 1: instructions may lead to incorrect medication use; error type 2: instructions for use are contradictory or vague; error type 3: there are factual (nonmedical) errors; error type 4: there is information unrelated to the prescription or that is entirely nonsensical (model hallucination).

**Table 2:** Composition of the weighted overall score for the evaluation of texts generated by the models.

Criterion	Weight	Number of items × value per item	Total achievement
Adequacy and correctness	2	2×4	8×2=16
Clarity	1.5	2×4	8×1.5=12
Usefulness	1.5	2×4	8×1.5=12
Completeness	1	2×4	8×1=8
Language simplification	1	2×4	8×1= 8

#### Data Collection

The distribution of the 48 test options, generated from 16 clinical scenarios and the 3 models, was randomly automated within the simulation environment. Each health care professional received at least 3 clinical scenarios that required the prescription of a medication. Upon completion of the prescription data, the usage instructions were automatically generated by 1 of the 3 models. The health care professional was unaware of which model generated the instructions presented sequentially after the prescription they created in the simulation environment.

### Outcomes and Data Analysis Methods

The primary outcome of this study was the weighted overall score achieved by the models, as assessed by evaluators using quantitative metrics, and for each specific criterion. The values for the positive and negative questions, as well as the specific scores achieved for the criteria of adequacy, completeness, clarity, language simplification, and usefulness, were compared among the 3 models. The secondary outcome focused on the incidence of errors identified within the texts generated by the models. Consequently, the models’ performance evaluation encompassed both the global score and the frequency of errors in the generated instructions.

Qualitative variables were described using absolute and relative frequencies. Measures of central tendency (average and median) and variability (SD or IQR or range) were calculated for quantitative variables, according to their distribution. To analyze parametric distribution, averages were compared using the ANOVA 1-way test, and for nonparametric distribution medians were compared using the Kruskal-Wallis test, since each instruction was independently generated, even using the same clinical scenario. We used Bonferroni post hoc test to compare subgroups. The consistency analysis of each criterion used in the scale consisted of Cronbach α. We assessed observer variability among health professionals for each simulated scenario and model, calculating the coefficient of variability, taken in percentual [20]. Statistical tests were conducted using SPSS software (version 26.0; IBM Corp). The significance level for hypothesis tests was 5%, and 95% CIs were used.

### Ethical Considerations

The study was approved by the Research Ethics Committee of the Federal University of Minas Gerais (CAAE: 78883924.7.0000.5149). The volunteers signed an informed consent form and did not receive any benefit for participating in our study.

## Results

### Overview

**Figure 4:**
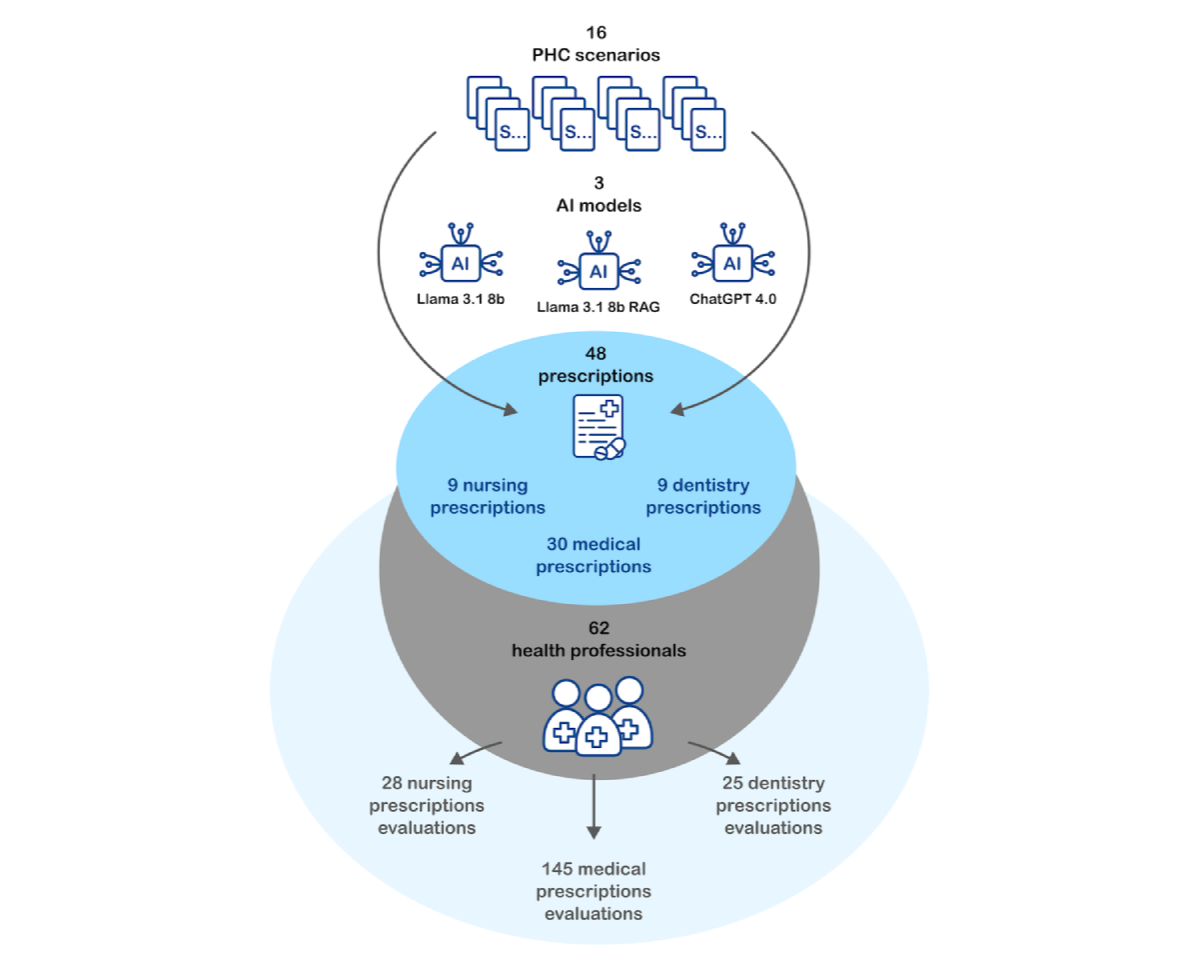
Distribution of large language model instructions evaluations performed on prescriptions prepared by health care professionals. AI: artificial intelligence; PHC: primary health care.

From the prescriptions prepared by health care professionals in the simulation environment, 198 evaluations of the performance of 3 models were conducted, distributed as shown in Figure 4.

### Model Performance by Clinical Scenario

Median of overall scores of 198 evaluations performed by 62 health professionals for 16 clinical scenarios is presented in Table 3. The instructions generated by ChatGPT-4.0 had overall scores above 90 in 4 (26.7%) and above 80 in 12 (80%) of 15 evaluated scenarios. The instructions generated by Llama3.1-8B models had overall scores above 90 in 3 (18.8%), and above 80 in 8 (50%) of 16. When generated by Llama3.1-8B-RAG, the instructions had values above 90 in 5 (33.3%), and above 80 in 9 (60%) of 15 scenarios. Interrater variability of scores among PHC professionals revealed wide differences of opinions ranging from 4.2% (n=3) to 85.8% (n=6).

**Table 3:** Model performance in the proposed clinical scenarios, by scenario (N=198). Values represent weighted scores based on the original 1-5 Likert scale.

Scenario	PHC^a^ professional	Received evaluations in ech scenario, n		Model performance (overall weighted scores)																	
				ChatGPT-4.0						Llama3.1-8B						Llama3.1-8B-RAG^b^					
				Model evaluations in each scenario, n		CV^c^ (%)		Median (IQR^d^)		Model evaluations in each scenario, n	CV (%)		Median (IQR)		Model evaluations in each scenario, n		CV (%)		Median (IQR)		
1	Dentist	9		3		26.6		85.7 (36.6)		3	4.2		100 (7.1)		3		13.2		85.7 (22.3)		
2	Dentist	9		3		60.4		84.8 (74.1)		3	7.7		88.4 (12.5)		3		25.1		85.7 (39.3)		
3	Dentist	7		3		9.8		85.7 (16.1)		2	13.7		91.1 (17.9)		2		7.3		95.1 (9.8)		
4	Nurse	16		4		12.6		90.6 (20.5)		6	5.3		95.5 (11.6)		6		37.2		86.2 (69.6)		
5	Nurse	7		4		25.5		86.6 (42.0)		3	78.6		26.8 (26.8)		0		NE^e^		NE		
6	Nurse	5		1		N/A^f^		65.2 (N/A)		1	N/A		82.1 (N/A)		3		20.9		93.8 (N/A)		
7	Physician	26		9		32.5		87.5 (50.0)		9	53.5		64.3 (83.9)		8		32.5		71 (65.2)		
8	Physician	28		9		24.0		89.3 (60.7)		10	57.3		40.2 (76.8)		10		26.3		67.9 (51.8)		
9	Physician	23		7		12.2		97.3 (31.3)		8	58.1		47.8 (90.2)		8		28.7		72.3 (80.4)		
10	Physician	22		8		31.8		90.6 (80.4)		7	36.2		83.9 (78.6)		7		41.3		83 (77.7)		
11	Physician	21		8		21.6		92.4 (50.0)		7	54.2		51.8 (71.4)		6		85.8		33 (95.5)		
12	Physician	4		0		N/A		NE		2	8.5		89.3 (10.7)		2		50.1		71.9 (50.9)		
13	Physician	5		2		11.9		84.8 (14.3)		1	N/A		60.7 (N/A)		2		34.4		66.1 (32.1)		
14	Physician	6		2		13.9		91.1 (17.9)		1	N/A		42.9 (N/A)		3		5.7		96.4 (10.7)		
15	Physician	7		2		15.1		75.0 (16.1)		2	18.9		70.1 (18.8)		3		2.5		92 (4.5)		
16	Physician	3		1		N/A		77.7 (N/A)		1	N/A		91.1 (N/A)		1		N/A		98.2 (N/A)		
Total by model			198		66		25.8		88.4 (22.8)	66		76.5		66.5 (50.9)		66		30.9		79.9 (24.7)	

^a^PHC: primary health care.

^b^RAG: retrieval-augmented generation.

^c^CV: coefficient of variation.

^d^IQR: interquartile range.

^e^NE: not evaluated.

^f^N/A: not applicable.

**Figure 5:**
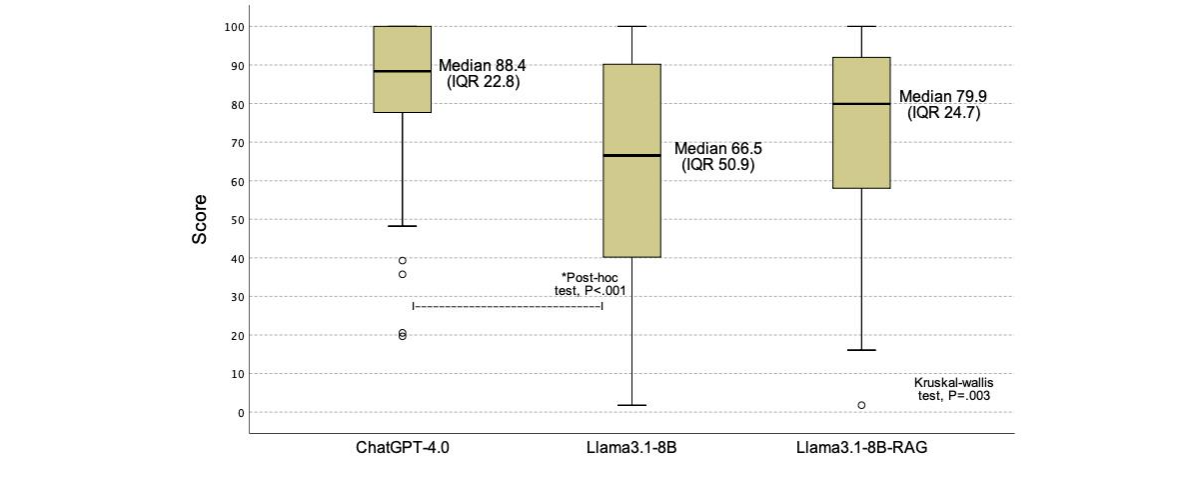
Comparisons of model performance in generating medication use instructions for digital prescriptions, according to health professionals’ assessment. IQR: interquartile range; RAG: retrieval-augmented generation.

Median values for the overall weighted score assigned to the 3 models across the scenarios were different among them (Kruskal-Wallis test, *P*=.003), as presented in Figure 5, with median (IQR) values. Llama3.1-8B-RAG received evaluations with similar overall scores to ChatGPT-4.0 (post hoc test *P*=.05) and similar to Llama3.1-8B (post hoc test *P*=.44). ChatGPT-4.0 outperformed Llama3.1-8B (Bonferroni test *P*<.001).

Upon separate and nonweighted evaluation of the 5 criteria that make up the overall score, there were differences among the models in all of the criteria, as presented in Table 4. Comparing pairs of models, the specific scores assigned to the models ChatGPT4.0 with the Llama3.1-8B-RAG, 4 criteria showed no differences: adequacy (post hoc test, *P*=.54), completeness (post hoc test *P*=.38), clarity (post hoc test *P*=.09), and usefulness (post hoc test *P*=.63), considering the total of positive and negative questions for each criterion. ChatGPT-4.0 received higher scores in the language simplification criterion, for both the positive (1-way ANOVA *P*=.001) and negative (1-way ANOVA *P*=.03) questions, as shown in Table 4. Llama3.1 without the RAG feature scored lower than ChatGPT-4.0 across all 5 criteria, matching ChatGPT-4.0’s results only for the language simplification—negative question “My prescription did not improve with the guidelines generated by the AI” (*P*=.06).

Internal consistency of the proposed scale had the Cronbach α value of 0.811 for adequacy and correctness, 0.641 for completeness, 0.757 for clarity, 0.680 for language simplification, and 0.724 for usefulness.

Regarding the presence or absence of errors, the 3 models were similar in terms of error type 1 (chi-square test *P*=.71), and type 4 (chi-square test *P*=.67), as presented in Table 5. ChatGPT-4.0 had a lower frequency of type 2, related to factual inconsistencies (chi-square test *P*<.001) and type 3, related to contradictory or vague instructions (chi-square test *P*=.02) errors. Llama3.1-8B-RAG had a lesser frequency of type 2 (n=11, 16.7%) and type 3 (n=5, 7.6%) errors compared to Llama3.1-8B without RAG, type 2 (n=23, 34.8%) and type 3 (n=10, 15.2%) errors.

**Table 4:** Specific and raw evaluation of adequacy, completeness, clarity, language simplification, and usefulness, by health care professionals (N=198)^a^.

Criteria			ChatGPT-4.0, mean (SD)		Llama 3.1- 8B, mean (SD)		Llama 3.1-8B-RAG^b^, mean (SD)		Comparisons among models^c^, *P* value					
									Overall		1	2		3
Positive question: the instructions are correct and consistent with widely accepted practices in the health care field.			4.45 (1.0)		3.80 (1.5)		4.23 (1.1)		.008		.006	.84		.13
Negative question: the instructions contain harmful or incorrect information regarding the use of medication.			1.64 (1.3)		2.41 (1.7)		1.98 (1.5)		.01		.008	.52		.29
**Adequacy and correctness: positive and negative sum**			6.82 (2.1)		5.39 (2.9)		6.24 (2.3)		.004		.003	.54		.14
	Positive question: the instructions covered relevant aspects for the correct use of the medication.	4.45 (0.9)		3.67 (1.4)		4.32 (1.0)		<.001		<.001		*>*.99	.03	
	Negative question: the instructions did not include sufficient information for the patient to correctly use the medication.	1.91 (1.4)		2.73 (1.6)		2.38 (1.5)		.008		.006		.22	.56	
**Completeness: positive and negative sum**			6.55 (1.8)		4.94 (2.7)		5.94 (2.2)		<.001		<.001	.38		.04
	Positive question: the instructions were sufficiently clear for the patient to correctly take or use the medication.	4.33 (1.0)		3.50 (1.5)		3.98 (1.3)		.001		.001		.35	.09	
	Negative question: the instructions present disorganized information that is difficult for the patient to understand.	1.65 (1.2)		2.56 (1.5)		2.21 (1.4)		.001		.001		.07	.46	
**Clarity: positive and negative sum**			6.68 (1.9)		4.94 (2.8)		5.77 (2.4)		<.001		<.001	.09		.13
	Positive question: the instructions were written in an accessible format and comprehensible to the patient.	4.64 (0.7)		3.62 (1.5)		3.83 (1.4)		<.001		<.001		.001	.93	
	Negative question: the instructions provided excessive technical information for the patient.	1.59 (1.2)		2.21 (1.4)		2.39 (1.6)		.003		.03		.003	>.99	
**Language simplification: positive and negative sum**			7.05 (1.5)		5.41 (2.5)		5.44 (2.6)		<.001		<.001	<.001		>.99
	Positive question: the suggested instructions were useful for complementing the text of the prescription I prepared.	4.30 (1.1)		3.64 (1.4)		4.05 (1.2)		.008		.006		.69	.17	
	Negative question: my prescription did not improve with the guidelines generated by the AI^d^.	2.35 (1.5)		2.97 (1.6)		2.62 (1.4)		.06		.06		.90	.55	
Usefulness: positive and negative sum			5.96 (2.2)		4.67 (2.7)		5.42 (2.4)		.01		.008	.63		.22

^a^Values represent raw scores based on the original 1-5 Likert scale.

^b^RAG: retrieval-augmented generation.

^c^ANOVA 1-way test. (1) Post hoc test ChatGPT-4.0 vs Llama3.1-8B. (2) Post hoc test ChatGPT-4.0 vs Llama3.1-8B-RAG. (3) Post hoc test Llama3.1-8B vs Llama3.1-8B-RAG.

^d^AI: artificial intelligence.

**Table 5:** Frequency of errors found in the instructions for use of medications generated by the models (N=198).

Frequency of errors	ChatGPT-4.0 (n^a^=66)	Llama3.1-8B (n=66)	Llama3.1-8B -RAG^b^ (n=66)	Comparisons between models^c^, *P* value
Error type 1: instructions leading to incorrect use of the medication, n (%) (95% CI)	15 (22.7) (13.8-33.8)	19 (22.8) (18.8-40.4)	19 (22.8) (15.0-35.4)	.71
Error type 2: contradictory or vague instructions for use, n (%) (95% CI)	4 (6.1) (1.9-13.5)	23 (34.8) (24.1-46.8)	11 (16.7) (9.0-26.8)	.001
Error type 3: factual errors, n (%) (95% CI)	1 (1.5) (0.1-6.5)	10 (15.2) (7.9-25.1)	5 (7.6) (2.8-15.6)	.02
Error type 4: there is information that is not related to the prescription, or that is completely meaningless (model hallucination), n (%) (95% CI)	7 (10.6) (4.7-19.5)	9 (13.6) (6.8-23.2)	6 (9.1) (3.7-17.6)	.67

^a^n: number of scenarios.

^b^RAG: retrieval-augmented generation.

^c^Chi-square test.

## Discussion

### Principal Findings

The adoption of technological approaches that support health care professionals at the point of care integrates the worldwide digital health landscape [21]. The most relevant finding of this study was demonstrating that LLMs performed well in generating supplementary instructions for medication prescriptions within PHC settings, as per health professionals’ evaluation. We consider this analysis essential to overcome the preclinical evaluation step of the solution, thereby qualifying the models for scalability planning.

In the development timeline, the 3 models were progressively adjusted to meet the demands for adequate and simplified medication use instructions. The model’s great overall scores, as attributed by the external evaluator panel, qualify the solution for a pilot implementation within the e-SUS PHC electronic health record environment. We highlight that the open-source Llama3.1-8B model was capable of performing the tasks defined in the prompt engineering, with or without RAG, with an overall quality equivalent to that of the closed-source ChatGPT-4.0 model, considered the state-of-the-art among LLMs [22]. The tailoring of messages in the context of health, by reducing jargon and vague terms and facilitating the comprehension of technical terms in the communication process between health care professionals and patients, is recognized as a promising frontier for generative AI [23].

The number of errors identified in the instructions generated by the models demonstrated the necessity for human oversight of the technology, which is expected in PHC settings in alignment with principles for the ethical and responsible use of AI [9]. For the introduction of this innovation in a real-world setting, we planned that health care professionals would be able to accept, edit, or delete the text suggested by LLM, taking responsibility for the final version presented to the patient. In this regard, both models demonstrated similar results, with fabricated information frequency ranging between 9.1% and 13.6%. These findings still require improvements in the technology, which is expected with an increased number of professionals using, fine-tuning, and evaluating the outputs.

The expected digital transformation in health care demands that systems offer functionalities centered on human values, prioritizing equity and privacy while addressing the challenges of care delivery. Usefulness, safety, and inclusion must be prioritized from the initial stages of AI development and scalability [20]. Reports on AI to promote treatment adherence are still incipient, however promising. In a systematic review, Reis et al [8] highlighted that AI-based interventions improve medication adherence by 6.7% to 32.7% compared to current practices in randomized clinical trials.

### Strengths and Limitations of the Study

The clinical evaluation of LLMs in health care settings is complex and demands interdisciplinary collaboration to meet both clinical requirements and rigorous validation criteria. A relevant point to qualify our findings is the diverse representativeness of PHC prescriber categories in the evaluation process, as highlighted in international consensus guidelines for the introduction of AI in health care [9]. Concerning the panel of evaluators, the majority of health care professionals expressed concerns about overreliance on the technology when using AI and patient safety. This reinforces the emphasis placed on the adequacy criterion in weighting the models’ performance score. Adequacy is a relevant criterion indicating the model’s capacity to generate responses appropriate to the purpose, context, and meet the patient’s needs. In the approach considering specific criteria, RAG added to Llama3.1-8B model proved sufficient to match the score attributed to ChatGPT-4.0 in the evaluation items of adequacy, completeness, clarity, and usefulness. However, RAG in PILs did not contribute to the language simplification of the guidance, which will require a prompt redesign.

Additionally, we emphasize the relevance of the randomized assignment of the PHC clinical scenarios and LLMs among evaluators, resulting in 46 different tests. These professionals were unaware of the prescription-inducing narratives and had no prior knowledge of the type of instructions the models might suggest during prescription preparation. In current practices, adding instructions for correct medication use to a prescription is not mandatory, nor is there a standard for the content of these instructions; both aspects are left to the prescriber’s discretion. Nonetheless, the majority of evaluators considered that LLM-generated instructions were adequate, complete, simplified, clear, and useful for complementing their prescriptions. It is relevant to note that during the first steps of the innovation’s development process, we defined the requirements for good practices on instructions, obtained through review of scientific articles and brochures [12]. We infer that LLMs will bring benefits to patients by facilitating their engagement in self-care through the correct use of prescribed medication. Although disagreements among human evaluators of generative AI outputs are expected [24], the majority of tests (n=38, 82.5%) in this study had scores attributed by more than one evaluator.

The results interpretation deserves some caution. We faced difficulties during the process of enrolling health professionals for the composition of the panel of evaluators. This challenge, joined with the random assignment of prescription scenarios, resulted in 2 out of the 48 elaborated tests having no evaluator and 8 (17.5%) with only 1 evaluator. Even in the absence of an established consensus in the literature regarding the ideal sample size for validation of generative AI systems with end users, our study achieved 62 volunteers with a diversity of PHC prescribers. Based on Holmes et al [25] findings, around 26 participants are needed to reveal usability flaws in chatbots, facing the open and nonlinear nature of natural language.

Regarding metrics of LLMs’ performance, the adaptation of the quality evaluation scale derived from the System Usability Scale was possible due to its generalizable structure [18]. Although originally proposed for interactive systems, the scale was adapted to specific criteria within the context of an e-prescribing system. The main advantages are that the polarity of positive and negative questions for each criterion demands more attention from the respondent and mitigates automatic or biased responses, increasing the reliability of the collected data. Beyond this, the overall weighted score transformation, which assigns importance to concepts and converts them into a standardized score on a scale of 0 to 100, enables numerical comparisons between different AI models, facilitating statistical analyses and enhancing evaluation based on performance criteria. Our metrics in scale were acceptable in terms of consistency, demonstrating potential for use as an initial and exploratory tool, since all of the criteria had Cronbach α values above 0.64 and 3 criteria above 0.72 [26]. Borderline domains, such as completeness and language simplification, should be reviewed and improved for adoption in our next implementation step, considering the real scenario evaluation. We state the lack of verification regarding differential effects on the instructions across groups of patients with varying educational levels, cultural aspects, age or health literacy, which demands further attention.

### Meaning and Generalizability of the Study

The implementation of AI in public health care settings faces several challenges, including the need for adequate infrastructure, secure and ethical access to data, ensuring privacy, disseminating technical knowledge for its sustainability, and establishing mechanisms for technological governance [23,27]. From the perspective of innovation scalability, the global weighted score of Llama 3.1-8B-RAG model was comparable to that of ChatGPT-4.0, except in terms of language simplification. Proprietary LLMs incur financial costs and send data to a web app, raising privacy concerns regarding sensitive health data. Open-source models are capable of local execution, making them viable and sustainable alternatives for public health deployment. Llama3.1-8B models demonstrated high performance when combined with RAG in PILs, which expands their capability by incorporating external information. The results pointed out a promising implementation, suggesting that other external information sources can enhance the model’s performance, such as language simplification, by allowing for a more detailed incorporation of patient data. Following the incorporation of technology into the national eSUS APS system, it will be possible to use data from the Brazilian citizens’ National Registry of SUS, which includes education, income, occupation, race, and gender. Even so, the use of LLMs in our innovation does not replace the individual attention of health care professionals with their patients, nor is it a solution for directly improving health literacy. However, it could improve the effective communication between PHC teams and their communities. A significant effect of language simplification of instructions on medication adherence requires repeated counseling sessions provided by health educators [28].

Another barrier to face is the provision of computational support that meets security, performance, and equity requirements for integrating AI processing into clinical workflows. The e-SUS PHC electronic health record is an example of an information system with a significant social role, aiming to adapt its interfaces and workflows to PHC work processes. It also considers the diversity of Brazilian municipalities, which are at unmapped stages of digital maturity [29].

Beyond infrastructure, the risk of transferring responsibility from the health care professional to technology must be considered. Comprehensive governance standards based on ethics, transparency, and model explainability can mitigate excessive dependence on technology while simultaneously contributing to overcoming resistance in the use of generative AI, directing the technology toward achieving greater efficiency in health care systems [22]. Efficiency, usefulness, and clinical impact on public health still need to be measured and monitored as requirements for reliable AI implementation in health care settings [9,22]. The expectation is that this innovation, integrated into the e-SUS PHC electronic health record, can contribute to greater adherence to medication treatment, promoting better health in alignment with the principles of universality and equity of the SUS.

### Conclusions

The results indicated the potential utility of LLMs in prescribing to complement prescriptions, contributing to patient clarity regarding correct medication use. Open-source models demonstrated similar performance to closed-source models, guiding the scalability phase toward an environment favorable to data privacy. The introduction of LLM-generated text into the e-prescribing workflow, even when well-designed with prompt engineering and enhanced with content based on PILs, necessitates validation by the prescribing professional to ensure safety and error control, as well as performance monitoring and governance.

## Appendix

Multimedia Appendix 1 Additional tables.

## Abbreviations

AI: artificial intelligence
ANVISA: Agência Nacional de Vigilância Sanitária, Health Surveillance Agency, Brazil
e-prescribing: electronic prescribing
e-SUS APS: e-SUS Primary Health Care
LLM: large language model
PHC: primary health care
PIL: patient information leaflet
RAG: retrieval-augmented generation
STARE-HI: Statement on Reporting of Evaluation Studies in Health Informatics
SUS: Unified Health System

## Data Availability

Data are available under the terms of the Creative Commons BY, which requires appropriate credit to the original creator when using or adapting the work. Data are deposited in publicly available repositories, as follows:

Data file 1 - Questionnaire (CC-BY): Data collection responses for characterization of health care professionals invited to the model. The anonymized database, in Portuguese, is available in the institutional repository of the Universidade Federal de Minas Gerais, Brazil [30].

Data file 2 - Evaluation (CC-BY): It contains the LLMs’ evaluation data, in Portuguese. Clinical scenarios are simulated. The performance of LLMs corresponds to the evaluator’s perception as defined by metrics: the adequacy model’s capacity to generate responses that are accurate and appropriate to the user’s purpose, context, and needs. Completeness: model’s capacity to generate responses or content that adequately covers all relevant aspects of a query or task. Clarity: model’s capacity to present information in an understandable, organized, and unambiguous manner. Language simplification: the model’s capacity to generate instructions that can be understood by the patient. Usefulness: model’s capacity to generate medication use instructions that are useful for complementing prescriptions. Error types: type 1: instructions may lead to incorrect medication use; type 2: instructions for use are contradictory or vague; type 3: there are factual (nonmedical) errors; type 4: there is information unrelated to the prescription or that is entirely nonsensical (model hallucination). The anonymized database is available at the institutional repository of the Universidade Federal de Minas Gerais, Brazil [31].
