# Supplementary material for: Large Language Model–Generated Patient Instructions for Prescriptions in Primary Health Care: Preclinical Algorithm Validation
Source: J Med Internet Res. 2026 May 26;28:e84444. doi: 10.2196/84444 (PMC13250494; doi:10.2196/84444)
Supplement: Multimedia Appendix 1 [file jmir_v28i1e84444_app1.docx]

**Large Language Model Generated Patient Instructions for Prescriptions in Primary Health Care: Preclinical Algorithm Validation**

Zilma Silveira Nogueira Reis, Reis, Z.S.N <[zilma.medicina@gmail.com](mailto:zilma.medicina@gmai.com)> ORCID 0000-0001-6374-9295

Elisa Tuler Albergaria, Albergaria, E.T <etuler@gmail.com > ORCID 0000-0003-3595-9978

Adriana Silvina Pagano, Pagano, A.S <apagano@ufmg.br> ORCID 0000-0002-3150-3503

Eura Martins Lage, Lage, E.M. <euramartinslage@gmail.com> ORCID 0000-0002-7614-695X

Flávia Ribeiro de Oliveira, <flaviagob@ufmg.br> ORCID 0000-0001-6282-7781

Cristiane dos Santos Dias, <profacristianedias@gmail.com> ORCID 0000-0001-6559-3300

Juliana Almeida Oliveira, Oliveira, J.A. < julianaoliveira_md@outlook.com> ORCID 0000-0002-4704-2318

Gláucia Miranda Varella Pereira, Pereira, G.M.V <glauciavarella@gmail.com> ORCID 0000-0003-4697-5295

Isaias Jose Ramos de Oliveira, <isaias.ramos@gmail.com> ORCID 0000-0001-9232-4309

Érico Franco Mineiro, <erico.acad@gmail.com>, ORCID 0000-0002-0602-1991

Igor Carvalho Lima Oliveira <igoroliveira128@hotmail.com>, ORCID 0009-0006-1005-3191

Davi dos Reis de Jesus, Reis, D. J.<davireisjesus@aluno.ufsj.edu.br> ORCID 0009-0000-7299-1542

Antônio Pereira de Souza Júnior, <antoniopereira@aluno.ufsj.edu.br> ORCID 0000-0001-5862-9272

Igor de Carvalho Gomes, <igor.gomes@saude.gov.br> ORCID 0000-0002-0883-0063

Rodrigo André Cuevas Gaete, <rodrigo.gaete@saude.gov.br> 0000-0002-8689-5428

Ricardo João Cruz-Correia, Cruz-Correia, R.J < ricardo.jc.correia@gmail.com > 0000-0002-3764-5158

Leonardo Chaves Dutra da Rocha, da Rocha, L.C.D <lcrocha@ufsj.edu.br> ORCID 0000-0002-4913-4902

**Table S1.** Data collection form on health care professionals invited to the model validation process.

| **Questions** | **Response** |
| --- | --- |
| Municipality where you work (and State) | Lists |
| Profession | Primary Health Care Nurse  Primary Health Care Dentist  Primary Health Care Doctor |
| Year of graduation in the above profession | Year |
| Sex/gender | Free text |
| Since when have you been using e-SUS APS (year you started using it)? | Year |
| What are your concerns about the introduction of artificial intelligence in healthcare (check all that apply)? | Patient safety;  Discrimination against minorities or disadvantaged people;  Impact on communication between humans;  None;  Other. |
| In your opinion, what are the potential disadvantages or risks of using artificial intelligence to support communication between the prescribing professional and the health system user? | Free text |
| In your opinion, what are the potential advantages of using artificial intelligence to support communication between the prescribing professional and the health system user? | Free text |

PHC: Primary Health Care.

**Table S2.** Simulated prescription-inducing scenarios used in the study validation environment.

| **Profile of the targeted prescriber** | **Context to stimulate prescribing in the test environment** | **Age (years)** | **Gender** | **Health Condition** | **Preparation** | **Continuous use** |
| --- | --- | --- | --- | --- | --- | --- |
| Dentist | You see a 43-year-old man at a Dental Specialty Center. You identify a gum abscess and begin treatment.  **Prescription Task**: Prescribe an analgesic medication to relieve pain, with clear instructions on how the person should use the medication. | 43 | M | Gum abscess | Tablet | No |
| Dentist | You are treating a 29-year-old woman presenting with pain and swelling following endodontic treatment. She is already using common analgesics.  **Prescription task:** Prescribe an anti-inflammatory medication with clear instructions for use. | 29 | F | Endodontic edema | Tablet | No |
| Dentist | You see a 62-year-old man. The diagnosis is acute periapical abscess. There is no history of allergies or comorbidities.  **Prescription Task**: Prescribe an antibiotic, with clear instructions for use. | 62 | M | Periapical abscess | Tablet | No |
| Primary Health Care Nurse | A young woman complained of discharge and a fishy vaginal odor. **Prescribing Task:** Following the vaginal discharge treatment protocol, prescribe metronidazole, with clear instructions on how the person should use the medication. | … | F | Vaginal discharge | Tablet | No |
| Primary Health Care Nurse | A 6-month-old baby is brought to the childcare clinic with iron deficiency anemia. **Prescription Task**: Prescribe treatment according to the Primary Health Care protocol, with clear instructions for use. | 0,5 |  | Anemia | Liquid | No |
| Primary Health Care Nurse | A 42-year-old man seeks consultation at the Primary Health Care Center with suspected dengue fever (fever, headache, retroocular pain and myalgia). **Prescription task:** Prescribe an antipyretic in accordance with Primary Health Care protocols, providing clear instructions for use of the medication. | 42 | M | Dengue | Tablet | No |
| Primary Health Care Doctor | 25-year-old male with mild asthma. **Prescribing Task**: Prescribe a bronchodilator, with clear instructions on how to use the medication. | 25 | M | Asthma | Inhalers | Yes |
| Primary Health Care Doctor | Male, 28 years old, diabetes mellitus.  **Prescription Task**: Prescribe NPH insulin, with clear instructions for use. | 28 | M | Diabetes Mellitus | Injection | Yes |
| Primary Health Care Doctor | Female, 18 years old, with mild atopic dermatitis.  **Prescription Task:** Prescribe a topical corticosteroid, with clear instructions for use. | 18 | F | Atopic dermatitis | Ointments | No |
| Primary Health Care Doctor | Male, 25 years old, with bacterial conjunctivitis.  **Prescription Task**: Prescribe antibiotic eye drops, with clear instructions for use. | 25 | M | Conjunctivitis | Drops | No |
| Primary Health Care Doctor | Male, 26 years old, with otitis externa.  **Prescription Task**: Prescribe a topical antibiotic, with clear instructions on how the person should use the medication. | 26 | M | Otitis | Orifice drops | No |
| Primary Health Care Doctor | Child, 10 years old, with head lice. **Prescription Task**: Prescribe topical treatment, with clear instructions on how to use the medication. | 10 |  | Pediculosis | Lotion | No |
| Primary Health Care Doctor | Pregnant woman, 27 years old, gestational age 28 weeks. Complains of vulvar itching and white, lumpy vaginal discharge. **Prescription Task**: Prescribe vaginal cream, with clear instructions on how to use the medication. | 27 | F | Vaginal discharge | Vaginal cream | No |
| Primary Health Care Doctor | Female, 40 years old, with altered laboratory findings of glycated hemoglobin (HbA1c < 7.5%) and fasting blood glucose, with a diagnosis of uncomplicated type 2 diabetes mellitus. She reports difficulties to improve lifestyle habits and has made several attempts in the past. **Prescription Task**: Prescribe an oral hypoglycemic agent with clear instructions for use. | 40 | F | Diabetes Mellitus | Tablet | Yes |
| Primary Health Care Doctor | Man, 50 years old, with systemic arterial hypertension and low cardiovascular risk.  **Prescription Task**: Prescribe antihypertensive medication, with clear instructions on how the person should use the medication. | 50 | M | Arterial hypertension | Tablet | Yes |
| Primary Health Care Doctor | Female, 40 years old, diagnosed with anxiety disorder.  **Prescription Task**: Prescribe an anxiolytic, with clear instructions for use. | 40 | F | Anxiety disorder | Tablet | Yes |

F: Female; M: Male

**Table S3.** Simulated scenarios prompting prescription, used in the study validation setting.

| **Medication according to the e-SUS PHC system** | **Option 1** | **Option 2** | **Option 3** |
| --- | --- | --- | --- |
| Dipyrone Sodium 1 g, tablet | paracetamol 500 mg, tablet | ibuprofen 600 mg tablet |  |
| Nimesulide 100 mg, tablet | ibuprofen 400mg, tablet | ketoprofen 150mg, capsule |  |
| Amoxicillin + potassium clavulanate 500 mg + 125 mg, tablet | amoxicillin 500 mg, capsule | benzathine penicillin 1,200,000 IU, powder for suspension for injection |  |
| Metronidazole 400mg, tablet | clindamycin phosphate 20 mg/g cream | metronidazole 100mg/g, gel |  |
| Ferripolimaltose 50 mg/ml, oral solution | ferrous sulfate (25 mg/ml elemental iron) 125 mg/ml, oral solution |  |  |
| Paracetamol 500 mg, tablet | paracetamol 750 mg, tablet | dipyrone sodium 500mg, tablet | dipyrone sodium 1g, tablet |
| Salbutamol sulfate 100 mcg/dose, Aerosol | fenoterol, hydrobromide 100 mcg/dose, Aerosol |  |  |
| Human insulin NPH 100iu/mL, solution for injection | regular human insulin 100iu/ml, solution for injection | insulin detemir 100iu/ml, solution for injection | insulin aspart 100iu/ml, solution for injection |
| Ciprofloxacin + hydrocortisone 2 + 10 mg/ml, vial | betamethasone dipropionate 0.5mg/g cream | clobetasol, propionate 0.5mg/g, cream |  |
| Gentamicin sulfate 5 mg/mL ophthalmic solution | tobramycin 0.3%, ophthalmic solution |  |  |
| Polymyxin B + Neomycin + Fluocinolone + Lidocaine 10,000 IU + 3.5 + 0.25 + 20 mg/mL, otological solution | ciprofloxacin + dexamethasone 3.5 + 1 mg/g, ointment |  |  |
| Permethrin 10mg/ml, shampoo | permethrin 10mg/ml, lotion | ivermectin 6mg, tablet |  |
| Miconazole, 2% nitrate, vaginal cream | clotrimazole 10mg/g, cream | nystatin 25,000 iu/g, vaginal cream |  |
| Metformin Hydrochloride 500mg, controlled release tablet | glibenclamide 5mg, tablet | empagliflozin 25mg extended release tablet | glimepiride 2mg, tablet |
| Enalapril Maleate 20mg, tablet | amlodipine besylate 5 mg tablet | losartan potassium 50mg, tablet |  |
| Clonazepam 2.5 mg/mL, oral solution | clonazepam 2mg, tablet | diazepam 5mg, tablet | lorazepam 2mg, tablet |

PHC: Primary Health Care.

**Table S4.** Characterization of prescription-prompting scenarios by variables.

| **Variable** | **Category** | **Statistic** |
| --- | --- | --- |
| Age (years), median (range) |  | 28.5 (16.5) |
| Gender, n (%) | Female | 6 (37.5) |
|  | Male | 8 (50.0) |
|  | Not specified for scenario | 2 (12.5%) |
| Indications for treatment (ICPC-2), n |  | 16 |
| Active ingredient, n |  | 16 |
| Frequency of use, n (%) | Continuous use | 5 (31.3) |
|  | Temporary use | 11 (68.7) |
| Preparation, n (%) | Aerosol | 1 (6.25) |
|  | Tablet | 8 (50.0) |
|  | Cream | 1 (6.25) |
|  | Injectable solution | 1 (6.25) |
|  | Ointment | 1 (6.25) |
|  | Ophthalmic solution | 1 (6.25) |
|  | Oral solution | 1 (6.25) |
|  | Oral suspension | 1 (6.25) |
|  | Otological suspension | 1 (6.25) |

International Classification of Primary Care-2 [https://www.sbmfc.org.br/ciap-2/].

**Table S5.** Resources used to generate instructions for use of the prescribed medication.

| **Input data** |
| --- |
| The process of automatically generating instructions for medication use begins with the collection of relevant data on the patient and the prescribed medication, following the standards adopted in the e-SUS APS electronic medical record system:   - First name of the patient. - Active ingredient of the medicine, concentration - Presentation form - Dose - Route of administration - Posology - Total prescribed quantity |
| **Requirements for instructions on use of medicines** |
| Based on input and consensus from healthcare experts, nine essential requirements for clear and personalized instructions were defined, ranging from the structure and clarity of instructions to specific aspects such as storage and administration times, available in the database [Protocols.IO](http://protocols.io) [dx.doi.org/10.17504/protocols.io.3byl49k92go5/v1](https://dx.doi.org/10.17504/protocols.io.3byl49k92go5/v1) . |
| **Base prompt used in models 1, 2 and 3** |
| 1. Specify the name of the medication and the form of presentation using numerical values ​​for doses and concentrations.  2. Use imperative for commands.  3. Provide instructions based on route of administration.  4. Follow the chronological order of the actions the patient must perform.  5. Mention any preparation steps before explaining how to administer the medication.  6. Indicate special storage requirements, if applicable.  7. Specify the frequency, time of day, and whether the medication should be taken with meals or daily events.  8. For long-term treatment, advise the patient to seek medical consultation before completing the prescribed treatment.  9. Instruct the patient to store the medication in a safe place, out of the reach of children, in the original packaging, and not to share it with other people. |
| **Automation procedures used in models 1, 2 and 3**   - To avoid ambiguity in the instructions for use of the medication to the patient, the generated text must be written in the imperative tense. - The specified route of administration and instructions should be arranged in chronological order. - To mitigate limitations of LLMs, such as difficulties in simple mathematical calculations, a suggested medication administration schedule table was incorporated to avoid conflicting instructions. - The need to renew the prescription was conditioned on the mention of an indefinite period of use in the e-SUS APS Electronic Medical Record. - A list of medications that interact with alcoholic beverages was prepared for internal consultation using the model, for which a standard warning phrase was included. - Requirements common to all prescriptions, such as storage guidelines, were prompted directly into the final model output, reducing the computational burden of LLM. |
| **Retrieval-Augmented Generation used in Model 2 (Llama3.1-8b-RAG)**   - The RAG technique allowed the models used to access updated information from drug leaflets at Anvisa to enrich the context and improve the quality of the instructions generated. - To ensure fast access to external content, an up-to-date database was developed containing medication leaflets extracted from the Anvisa website using web crawling techniques. - To minimize the high computational cost of unrestricted use of full-text drug leaflets from Anvisa and to reduce the likelihood of including irrelevant or overly technical information, an on-demand Retrieval-Augmented Generation (RAG) approach was adopted. This method retrieves only specific excerpts from the leaflet corresponding to the prescribed medication. - To achieve adequate performance and avoid information overload in the model, the selection of relevant content was optimized using the cosine distance. The leaflet was divided into vectorized blocks, identifying the section closest to the phrase "6. How should I use this medicine?". Present in a standardized form in most leaflets, this phrase introduces Section 6, which generally contains the instructions for use or dosage. - To simplify the technical language of drug leaflets, the extracted information was summarized by the model via a specific Prompt (i.e. Extract and summarize dosage). The information extracted from this step is added to the prompt that requests the generation of instructions. |

APS: Primary Health Care; Anvisa: Agência Nacional de Vigilância Sanitária. LLM: Large Language Model. RAG: Retrieval Augmented Generation.

**Table S6.** Assessment form used in the model validation process.

1. **Evaluator confidence**

I feel confident, with the necessary knowledge to act as an evaluator of this prescription simulation.

0 ------------------------------------------------------------------------------100

1. **In each prescription with instructions for use generated by the model**

For the statements below, choose the option that best represents your opinion:

|  |  | Strongly disagree  (1 point) | Somewhat Disagree  (2 points) | Neither agree or disagree  (3 points) | Somewhat agree  (4 points) | Strongly agree  (5 points) |
| --- | --- | --- | --- | --- | --- | --- |
| 1 | The instructions are accurate and consistent with widely accepted practices in the healthcare field. |  |  |  |  |  |
| 2 | The instructions contain harmful or incorrect information about the use of medications. |  |  |  |  |  |
| 3 | The instructions cover the aspects relevant to the correct use of the medication. |  |  |  |  |  |
| 4 | The instructions do not include sufficient information for the health system user to use the medication correctly. |  |  |  |  |  |
| 5 | The instructions are clear enough for the health system user to take/use the medication correctly. |  |  |  |  |  |
| 6 | The instructions present poorly organized information and are difficult for the person (user) to understand. |  |  |  |  |  |
| 7 | The instructions are written in an accessible and understandable way for the health system user . |  |  |  |  |  |
| 8 | The instructions provide the person (user) with excessively technical information. |  |  |  |  |  |
| 9 | The instructions are useful to complement the prescription text I have written. |  |  |  |  |  |
| 10 | My prescription did not improve with the AI-generated instructions. |  |  |  |  |  |

Order of questions in the form:: 1, 5, 9, 2, 7, 3, 10, 6, 8, 4

1. **If you found error(s), mark the type(s) of error detected:**

|  | Instructions may lead to incorrect use of this medicine |
| --- | --- |
|  | Usage instructions are contradictory or vague |
|  | There are factual (non-medical) errors |
|  | There is information that is not related to the prescription or is completely meaningless (model hallucination) |

**4. Comments (free text)**

|  |
| --- |

**Table S7.** Frequency of errors found in the instructions for use of medications generated by the models.

|  | **ChatGPT-4.0**  n=15 | **Llama3.1-8B**  n=16 | **Llama3.1-8B -RAG**  n=15 |
| --- | --- | --- | --- |
| **Error type 1:**  Instructions leading to incorrect use of the medication, n, % (95% CI) | 3, 20%  (4.3 a 48.1%) | 4, 25%  (0.2 a 22.4%) | 2, 13.3%  (1.7 a 40.5%) |
| **Error type 2:**  Contradictory or vague instructions for use n, % (95% CI) | 0 | 5 (31.3)  (11.0 a 58.7%) | 1, 6%  (0.2 a 32%) |
| **Error type 3:**  Factual errors n, % (95% CI) | 0 | 1, 6.3%  (0.2 a 30.2%) | 1, 6.7%  (0.2 a 32%) |
| **Error type 4:**  There is information that is not related to the prescription or that is completely meaningless (model hallucination) n, % (95% CI) | 2, 13,3%  (1,7 a 40,5%) | 1, 6.3%  (0.2 a 30.2%) | 0 |

n= number of scenarios. RAG: Retrieval Augmented Generation.

**Table S8.** Coordinates of the ROC curve with values ​​of the global score achieved by all models, to discriminate errors.

| Positive if greater than or equal to^a^ | Sensitivity | 1 - Specificity |
| --- | --- | --- |
| 17,800 | 1,000 | 1,000 |
| 25,250 | 1,000 | ,929 |
| 35,500 | 1,000 | ,857 |
| 41,100 | 1,000 | ,786 |
| 47,100 | 1,000 | ,643 |
| 56,000 | 1,000 | ,571 |
| 62,950 | 1,000 | ,500 |
| 65,650 | 1,000 | ,429 |
| 68,100 | ,969 | ,429 |
| 71,000 | ,969 | ,357 |
| 72,550 | ,969 | ,286 |
| 74,100 | ,938 | ,286 |
| 75,450 | **,906** | **,214** |
| 76,350 | ,875 | ,214 |
| 77,250 | ,844 | ,214 |
| 79,900 | ,813 | ,143 |
| 83,000 | ,781 | ,143 |
| 84,350 | ,750 | ,143 |
| 85,050 | ,719 | ,071 |
| 85,500 | ,688 | ,071 |
| 86,600 | ,656 | ,071 |
| 88,400 | ,625 | ,071 |
| 90,200 | **,594** | **,000** |
| 91,550 | ,500 | ,000 |
| 93,300 | ,406 | ,000 |
| 94,850 | ,344 | ,000 |
| 95,300 | ,313 | ,000 |
| 95,750 | ,281 | ,000 |
| 96,650 | ,250 | ,000 |
| 97,750 | ,188 | ,000 |
| 98,450 | ,156 | ,000 |
| 99,350 | ,094 | ,000 |
| 101,000 | ,000 | ,000 |

The test outcome variable or variables: SUS_Weighted have at least one tie between the positive actual state group and the negative actual state group.

^a^ The lowest cutoff value is the minimum observed test value minus 1, and the highest cutoff value is the maximum observed test value plus 1. All other cutoff values ​​are the

means of two consecutive ordered observed test values.

**Table S9.** Assessment of the adequacy, completeness, clarity, personalization and usefulness of the instructions generated by the models, from the perspective of health professionals (n=192).

|  | **ChatGPT 4.0**  median (IIQ) | **Llama3.1 8B**  Median (IIQ) | **Llama3.1 8B-RAG**  median (IIQ) | **Comparisons between models*** |
| --- | --- | --- | --- | --- |
| **Adequacy** (weight 2) | 16 (2) | 15 (7.8) | 15 (3) | *P*=.129 |
| **Completeness** (weight 1) | 7 (2) | 6 (4.1) | 7 (3) | *P*=.014** |
| **Clarity (**weight 1,5) | 10,5 (1.5) | 10,5 (7.5) | 10,5 (3) | *P*=.070 |
| **Personalization** (weight 1) | 8 (1) | 6 (3.4) | 6,5 (3) | *P*=.126 |
| **Usefulness** (weight 1,5) | 10,5 (1.5) | 8,6 (5.8) | 9 (3) | *P*=.177 |

*Friedman paired test. IIQ: Interquartile range

**Post-hoc test: In the comparison Llama3.1 8B-RAG vs. Llama3.1 8B, *P*=.036. In the comparison ChatGPT 4.0 vs Llama3.1 8B, *P*=.011.

RAG: Retrieval Augmented Generation.

Note: The score differences were statistically similar for the dimensions of Adequacy, Clarity, Personalization, and Usefulness. A significant difference was observed in the Completeness dimension, with Llama3.1 8 B-RAG outperforming Llama3.1 8B (*P*=.036). ChatGPT 4.0 performed significantly better than Llama3.1 8B (*P*=.011) and showed comparable performance to Llama3.1 8 B-RAG.
